# Concomitant Convergent Ablation, Left Atrial Appendage Closure, and Minimally Invasive Direct Coronary Artery Bypass: A Case Series

**DOI:** 10.7759/cureus.95372

**Published:** 2025-10-25

**Authors:** Frederik C Loft, Sune Damgaard, André M Korshin, Christian L Carranza

**Affiliations:** 1 Department of Cardiothoracic Surgery, Copenhagen University Hospital - Rigshospitalet, Copenhagen, DNK; 2 Department of Thoracic Anaesthesiology, Copenhagen University Hospital - Rigshospitalet, Copenhagen, DNK

**Keywords:** atrial fibrilation, convergent procedure, coronary artery disease, epicardial ablation, left atrial appendage occlusion, midcab, minimally invasive procedures

## Abstract

We present the first report of concomitant convergent ablation, left atrial appendage (LAA) closure, and minimally invasive direct coronary artery bypass (MIDCAB) procedures during a single operation. Three patients had coronary artery disease of the left anterior descending artery and atrial fibrillation. All were offered concomitant convergent ablation and LAA closure during the MIDCAB procedure. There were no intra- or postoperative events. At one-year follow-up, no serious adverse events had occurred. One patient had experienced a relapse of paroxysmal atrial fibrillation, and one patient, with a well-functioning graft, had been medically optimized for angina. Our report shows that combining convergent ablation, LAA closure, and MIDCAB can be done feasibly and safely, enabling efficient minimally invasive approaches to future complex cases.

## Introduction

Minimally invasive cardiac surgery is used to reduce the surgical trauma in patients [1]. In atrial fibrillation, the Cox-Maze III/IV procedure has shown a high success rate of maintaining sinus rhythm, but at the cost of sternotomy and cardiopulmonary bypass [2]. Thus, minimally invasive surgical and catheter-based methods have been developed. The minimally invasive hybrid convergent procedure combines epicardial and endocardial catheter ablation for persistent atrial fibrillation, with increased efficacy compared to solitary endocardial catheter ablation [3]. Due to the altered blood flow in atrial fibrillation, the left atrial appendage (LAA) becomes a source of clot formation with the risk of stroke and embolism [4]. Thoracoscopic closure of the LAA is a safe and effective minimally invasive method for preventing stroke in atrial fibrillation [4]. For stenoses of the left anterior descending artery (LAD) not optimal for percutaneous coronary intervention (PCI), the minimally invasive direct coronary artery bypass (MIDCAB) procedure has proven safe and efficient for left internal mammary artery (LIMA) to LAD grafting [5]. Combining minimally invasive procedures can create new approaches for complex patients while still limiting the surgical trauma.

The aim of this study was to assess the feasibility and safety of concomitant convergent ablation, LAA closure, and MIDCAB during a single-stage operation. In this article, we present the first three cases performed at our institution, and to the best of our knowledge, this is the first report of these combined procedures.

## Case presentation

Patient selection

All patients were first scheduled for the MIDCAB procedure at the coronary multidisciplinary team conferences. The patients were selected for MIDCAB in cases of complex or diffusely diseased LAD not amenable to PCI, provided that the vessel was angiographically deemed graftable. In cases of two-vessel disease, the left circumflex artery (LCx) or right coronary artery had to be suitable for subsequent PCI; otherwise, the patients were referred for conventional coronary artery bypass grafting (CABG). Afterwards, due to atrial fibrillation, the patients were offered concomitant convergent ablation and LAA closure during the MIDCAB procedure. Potential risks of combining the procedures included prolonged anesthesia, cumulative surgical trauma with increased bleeding risk, and augmented postoperative pain. These factors were carefully monitored and managed intra- and postoperatively.

Patient A

**Figure 1:**
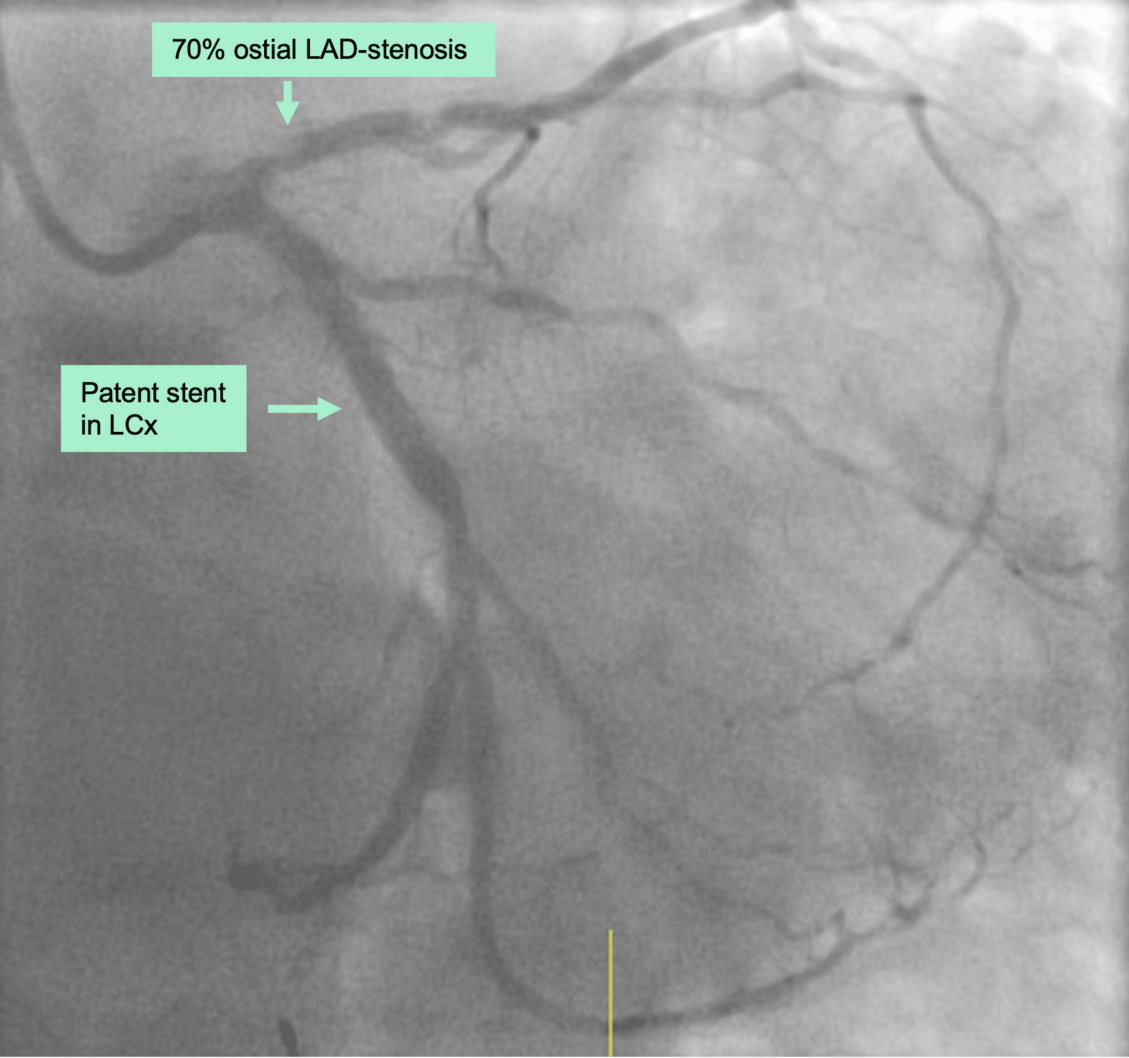
Preoperative coronary angiography of patient A showing a patent stent of LCx, but a 70% stenosis of the ostial LAD. LCx: left circumflex artery; LAD: left anterior descending artery.

A 72-year-old man underwent PCI with rotablation of the LCx one year prior to surgery (Table 1). Three months before surgery, a coronary angiography showed a patent stent, but 70% stenosis of the ostial LAD (Figure 1). Besides a previous medical history of hypercholesterolemia, asthma, and smoking, the patient also had paroxysmal atrial fibrillation (PAF), diagnosed five years prior to surgery. Treatment consisted of metoprolol 75 mg and apixaban 5 mg, both twice daily. The patient had no chest pain or palpitations, but dyspnea during physical exercise (Canadian Cardiovascular Society angina class [CCS] 0 and New York Heart Association functional class [NYHA] II), and a preoperative echocardiography showing an ejection fraction of 60% and no valvular disease.

**Table 1:** Baseline characteristics and outcomes AF = atrial fibrillation; CCS = Canadian Cardiovascular Society angina class; EF = ejection fraction; HF = heart failure; LAD = left anterior descending artery; LCx = left circumflex artery; NYHA = New York Heart Association functional class; PAF = paroxysmal atrial fibrillation; PCI = percutaneous coronary intervention; RFA = radiofrequency ablation; WPW = Wolff-Parkinson-White syndrome.

	Patient A	Patient B	Patient C
BASELINE CHARACTERISTICS			
Age, years	72	64	76
Sex	Male	Male	Male
Previous PCI	LCx, with rotablation (1 year before)	None	None
Coronary angiography	70% ostial LAD	80% ostial LAD + 70% peripheral LAD	75% mid LAD + 85% peripheral LAD, 70% mid LCx
Atrial fibrillation	PAF, diagnosed 5 years prior	PAF, RFA 11 months prior, recurrent	New-onset AF with HF
CCS class	0	III	0
NYHA class	II	II–III	II
Pre-op EF (%)	60	60	40 (after optimization, initial 15)
Valvular disease	None	None	None
Comorbidities	Hypercholesterolemia, asthma, smoking	WPW ablation, prior stroke, hypertension, hypercholesterolemia	Heart failure, prior stroke, hypertension, hypercholesterolemia
OUTCOMES			
In hospital	PAF	No adverse events	No adverse events
3-month follow-up	Asymptomatic, ECG showing sinus rhythm	Asymptomatic, ECG showing sinus rhythm	Asymptomatic, ECG showing sinus rhythm
12-month follow-up	PAF	Angiography without the need for stenting	No adverse events

Patient B

**Figure 2:**
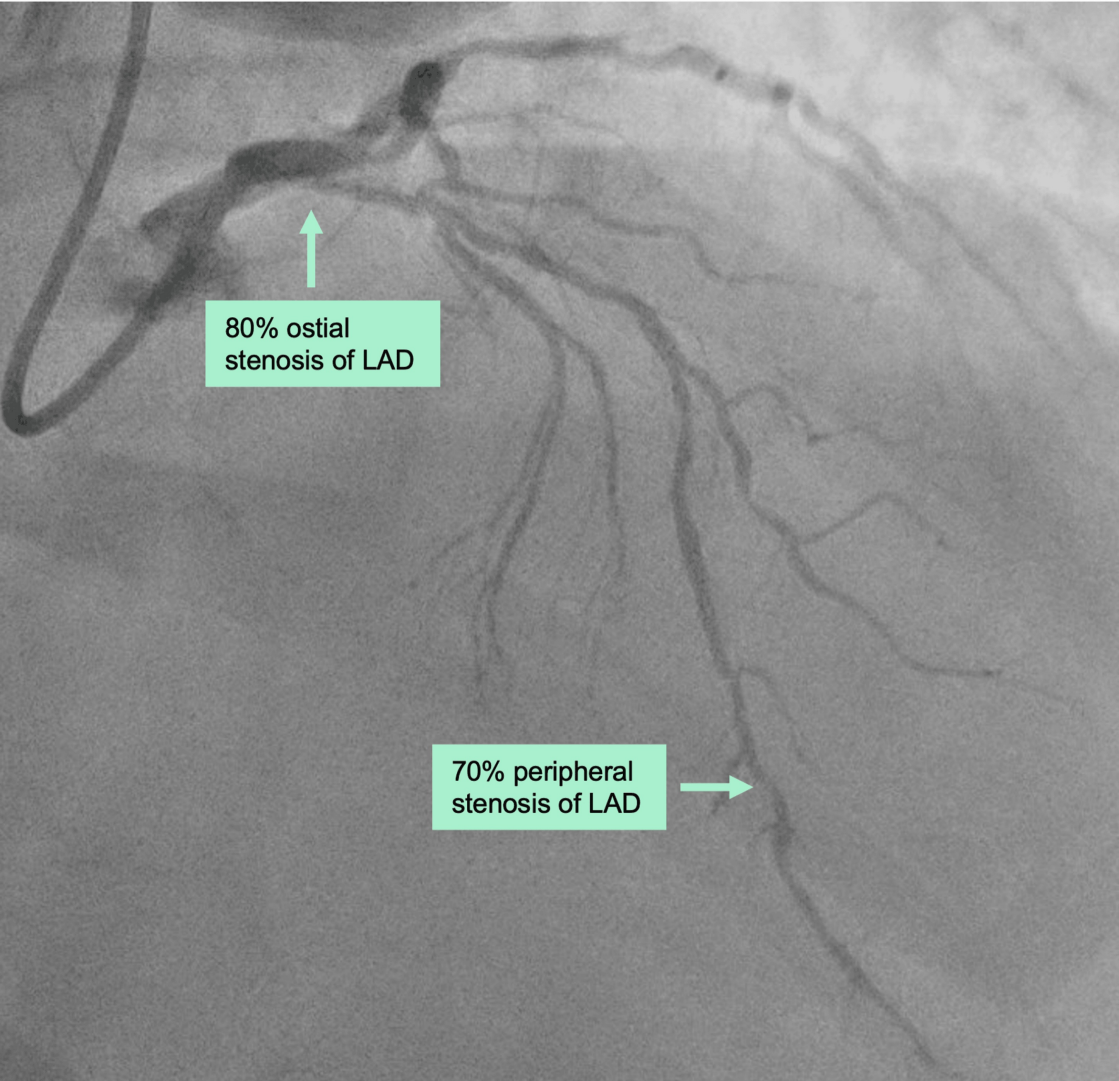
Preoperative coronary angiography of patient B showing an ostial 80% stenosis and a peripheral stenosis of 70% of the LAD. LAD: left anterior descending artery.

A 64-year-old man suffered stable angina pectoris, exercise-induced dyspnea, and dizziness (NYHA II-III and CCS III) and had an ostial 80% stenosis and a peripheral stenosis of 70% of the LAD (Table 1, Figure 2). The patient had PAF and received radiofrequency catheter ablation (RFA) 11 months prior to surgery, but subsequently had two hospital admissions due to symptomatic PAF. The patient was medicated with metoprolol 50 mg twice daily and rivaroxaban 20 mg once daily. The patient's comorbidities included hypertension, hypercholesterolemia, previous stroke, and previous endocardial catheter ablation for Wolff-Parkinson-White syndrome. Preoperative echocardiography showed an ejection fraction of 60% and no valvular disease.

Patient C

**Figure 3:**
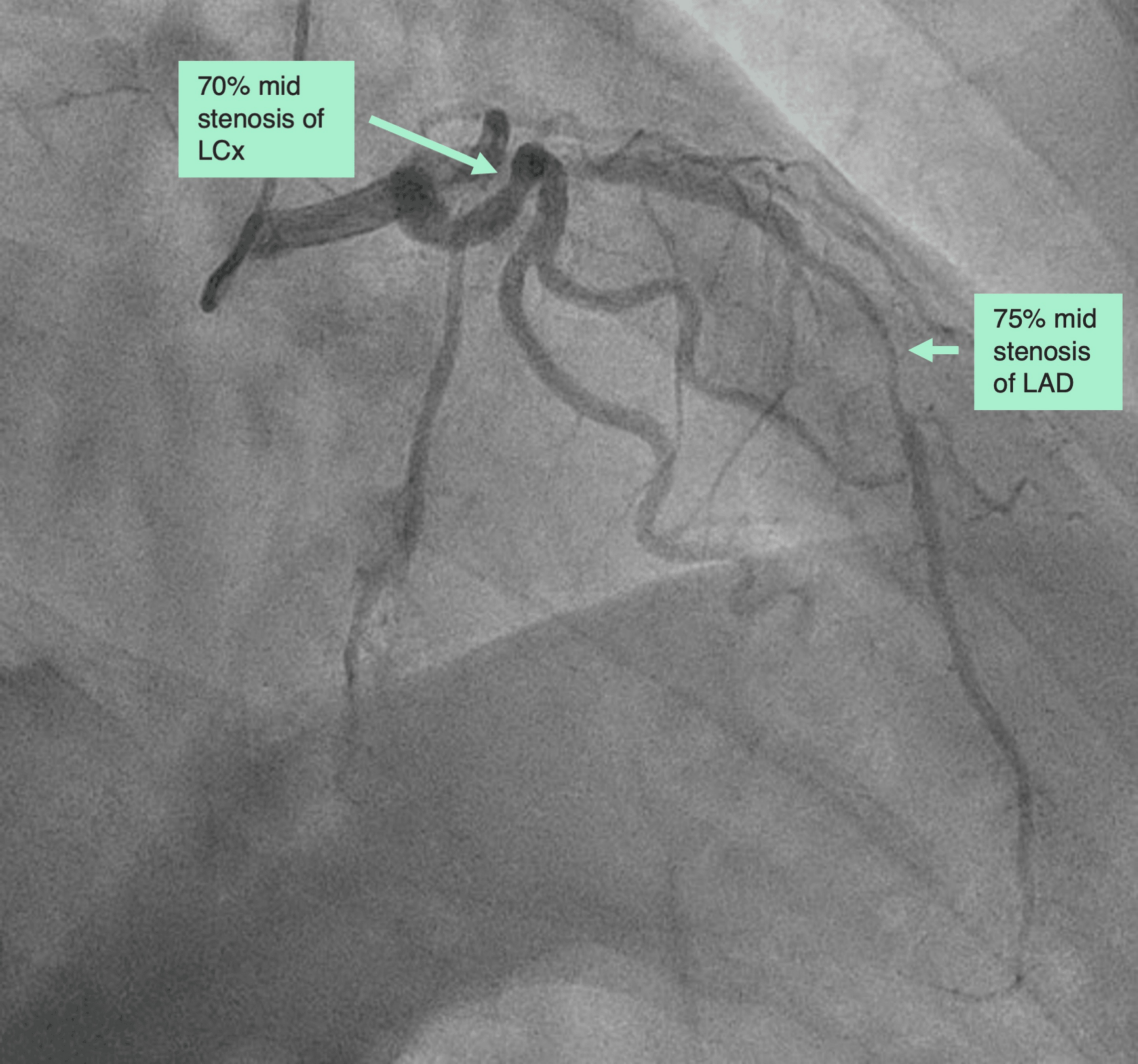
Preoperative coronary angiography of patient C showing a 75% mid stenosis of LAD and a 70% mid stenosis of LCx (85% peripheral stenosis of LAD not shown). LCx: left circumflex artery; LAD: left anterior descending artery.

A 76-year-old man was admitted with new-onset heart failure (ejection fraction of 15%) and rapid atrial fibrillation (Table 1). Through decongestion and rate control, the patient was optimized to an ejection fraction of 40% and a controlled heart rate. The patient then went through an elective direct current (DC) cardioversion and a coronary angiography showing 75% mid stenosis and 85% peripheral stenosis of LAD and a 70% mid stenosis of LCx (Figure 3). The patient had mild dyspnea and no chest pain (NYHA II and CCS 0), and preoperative echocardiography showed an ejection fraction of 40% and no valvular disease. The patient also suffered from hypertension, hypercholesterolemia, and a previous stroke and was treated with carvedilol 12.5 mg twice daily, apixaban 5 mg twice daily, valsartan and sacubitril 103 mg + 97 mg twice daily, dapagliflozin 10 mg once daily, and spironolactone 50 mg once daily.

All patients were offered concomitant convergent ablation and LAA closure during the MIDCAB procedure. Due to the 70% mid stenosis of LCx, patient C was preoperatively also scheduled for PCI of LCx on postoperative day 3, in addition to the MIDCAB. Patients were to be referred to endocardial ablation at three months of follow-up if symptomatic of atrial fibrillation.

Surgical technique

**Figure 4:**
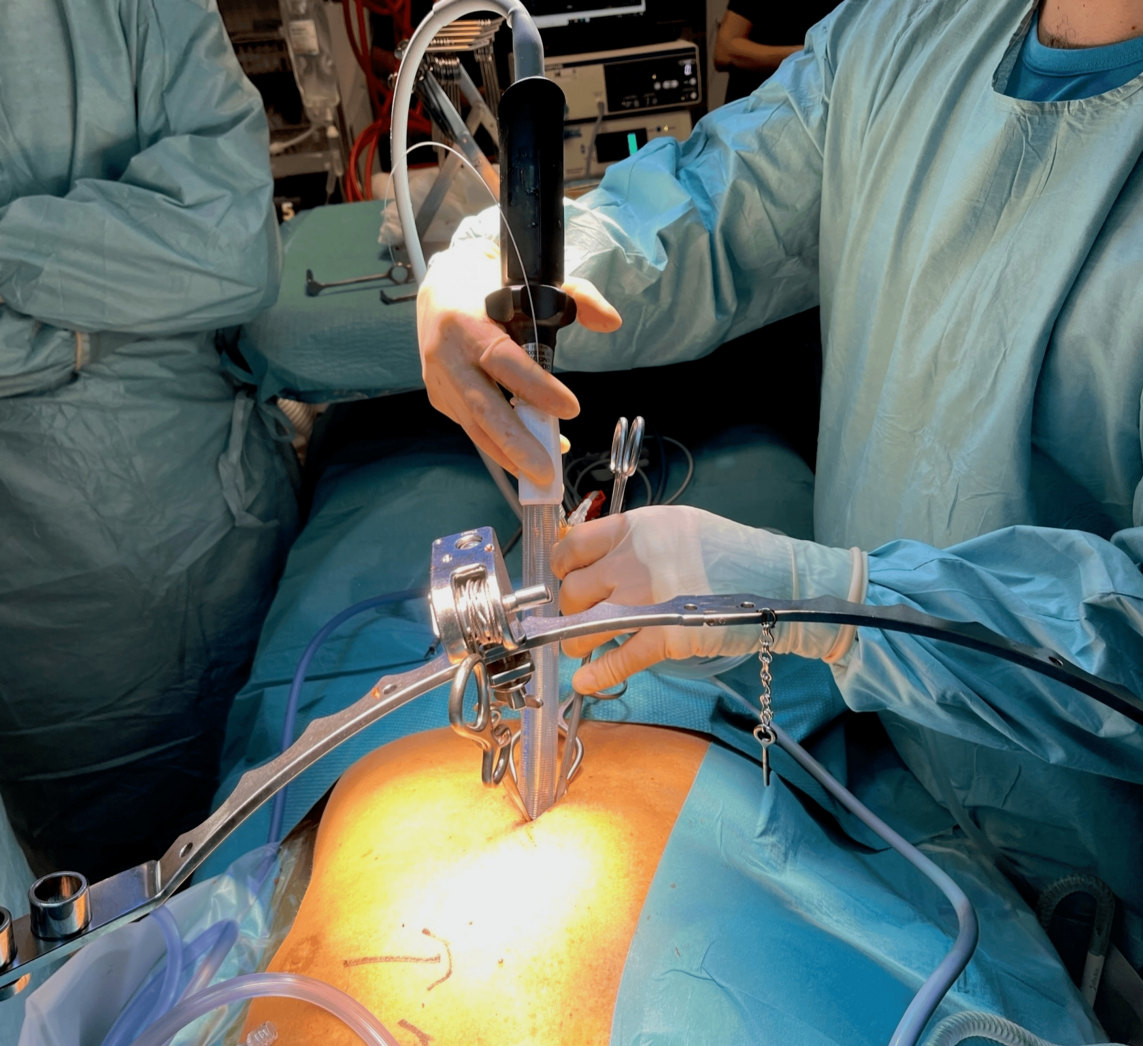
The subxiphoid access for the convergent ablation of patient B. Through the pericardioscopic cannula, the radiofrequency ablation device and the endoscope enable ablation of the posterior wall of the left atrium and the inferior pulmonary veins.

The convergent ablation was performed first to enable the water-cooling system to function in the closed pericardium and avoid damage to the coronary anastomosis. A 12-lead temperature probe was placed in the esophagus guided by X-ray. Through a subxiphoid access, the radiofrequency ablation device (Epi-sense, AtriCure, Mason, OH, USA) and a 5 mm 30° endoscope were placed inside the pericardioscopic cannula (Figure 4). Epicardial ablation was performed at the posterior wall of the left atrium and the inferior pulmonary veins.

**Figure 5:**
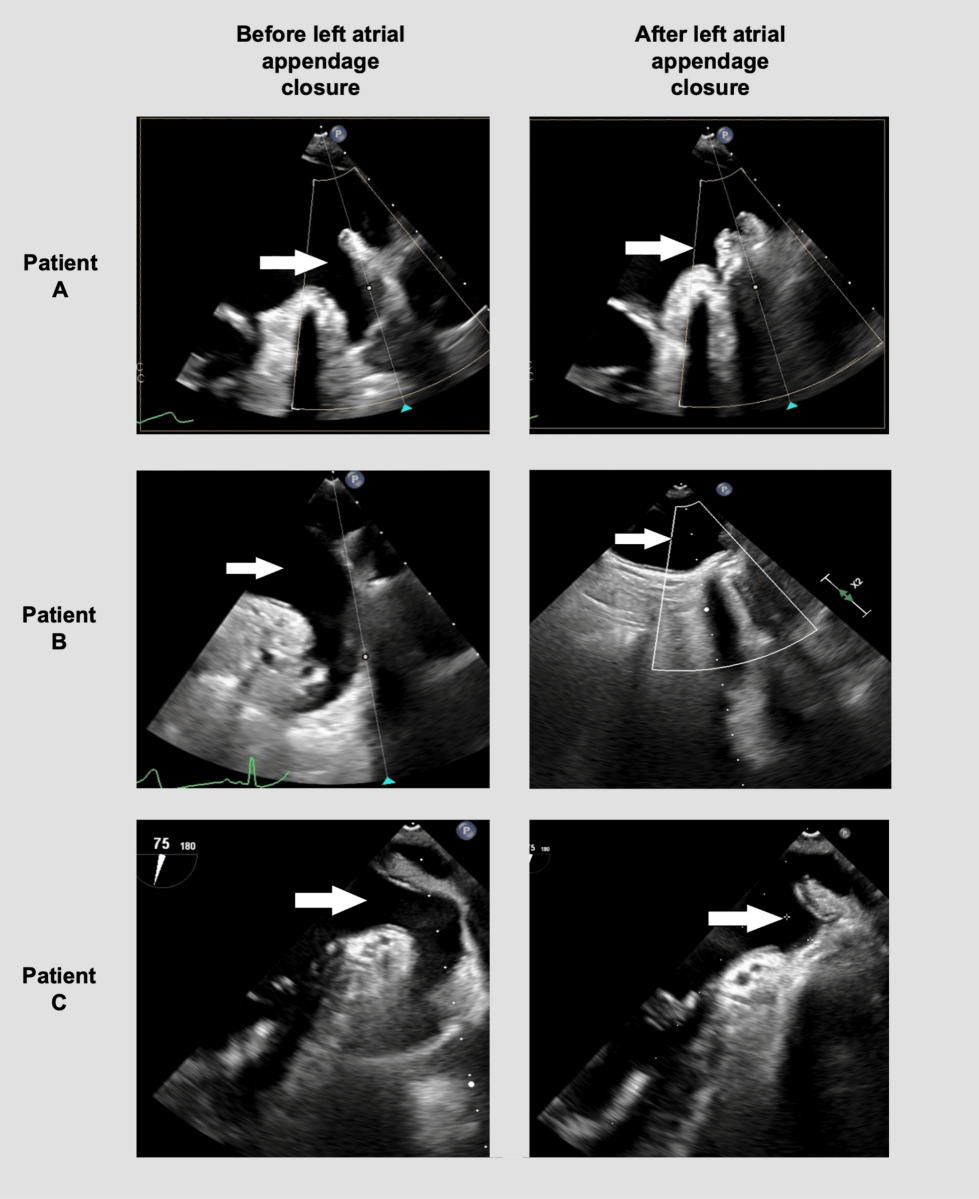
Intraoperative transesophageal echocardiography before and after the left atrial appendage closure of all patients. White arrows mark the left atrial appendage.

Afterwards, the MIDCAB procedure was performed video-assisted thoracoscopically using three ports. At first, the LIMA was harvested using a diathermy hook and the Harmonic Ultracision (Ethicon, Cincinnati, OH, USA). The pericardium was accessed, and the LAA was closed using AtriClip (AtriCure, Mason, OH, USA) (35 and 40 mm) (Figure 5).

**Figure 6:**
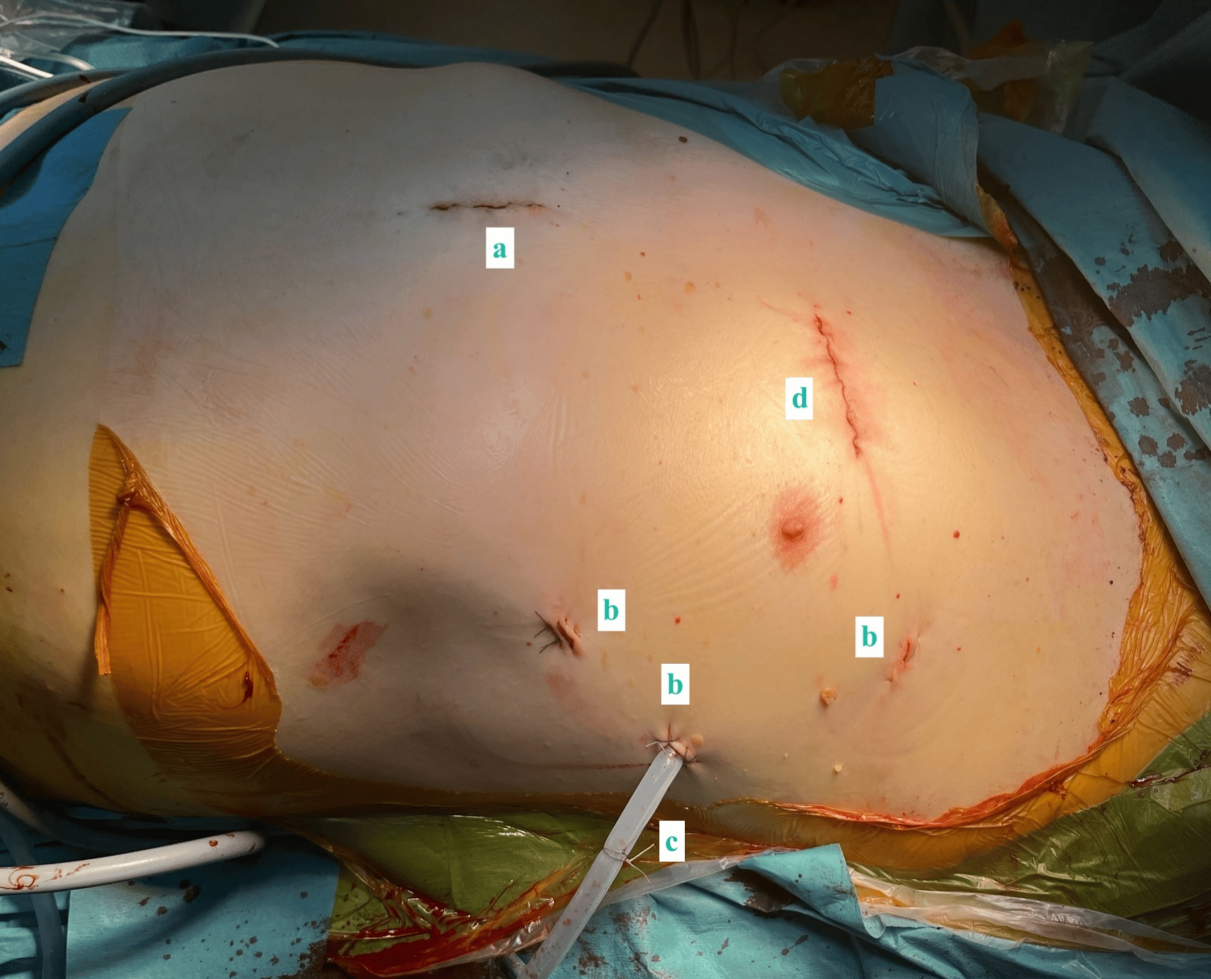
The postoperative surgical wounds of patient A, with preservation of the sternal bone. a) Subxiphoid access. b) Ports. c) Chest tube. d) Anterior thoracotomy.

Next, through a 5 cm anterior thoracotomy at the fourth intercostal space, the pericardium was opened, and the LIMA was grafted to the LAD using Octopus Nuvo (Medtronic, Minneapolis, MN, USA) and an intraluminal shunt. Afterwards, a chest tube was placed through one of the ports and the surgical incisions sutured (Figure 6). For the first case, a double-lumen tube was used, enabling deflation of the left lung during MIDCAB and LAA closure. In the second and third cases, CO_2 _expansion was used, with no change in the surgical conditions. The surgical procedures lasted 5 hours and 15 minutes, 4 hours and 5 minutes, and 4 hours and 29 minutes for patients A, B, and C, respectively.

Outcomes

All patients spent one night in our fast-track ward of the Intensive Care Unit and were transferred to our general ward the next morning. Patient A had postoperative PAF and was treated with amiodarone intravenously and orally and a reduced dose of metoprolol (50 mg twice daily). The patient had an unsuccessful DC cardioversion on postoperative day 2 but was discharged on day 4 in sinus rhythm. Patient B was discharged on day 4 in sinus rhythm and without any adverse events. Both patients had echocardiography performed at discharge, showing normal ejection fraction and no pericardial effusion. Patient C had no adverse events and was discharged on day 4 in sinus rhythm. Patient C was preplanned for PCI of LCx, but the coronary angiography during the procedure showed a well-functioning LIMA-LAD and a fractional flow reserve of 0.89 in LCx, and therefore, no need for stenting. Due to a new protocol, where minimally invasive patients undergoing non-valvular surgery do not require a routine postoperative echocardiography, patient C did not receive an echocardiography prior to discharge.

At three months follow-up, all patients were in sinus rhythm assessed by 12-lead ECG and reported no symptoms of arrhythmia since the procedure, including dyspnea, fatigue, and palpitations, whether intermittent or persistent. At one-year follow-up, patient A had experienced an episode of PAF recurrence and had been referred for electrophysiological mapping/endocardial complementary ablation. At one-year follow-up, patient B was in sinus rhythm but had undergone angiography due to angina. A well-functioning LIMA-LAD was found, but also diffuse coronary disease. Anti-anginal medication was optimized. At one-year follow-up, patient C was in a stable sinus rhythm and without symptoms of ischemia.

## Discussion

Our report shows that concomitant convergent ablation, thoracoscopic LAA closure, and MIDCAB procedures during a single operation are safe and feasible at one year of follow-up. The hybrid convergent procedure is mainly indicated for persistent or long-standing persistent atrial fibrillation but has reduced arrhythmia recurrence in PAF and was thus offered to our patients [6]. Combining MIDCAB and epicardial ablation has previously been performed, but this is the first report using the simpler and safer convergent procedure [3,7,8]. Results indicate that MIDCAB needs fewer revascularizations compared to PCI, and the convergent procedure is more effective than regular endovascular ablation, and thus a combination of these could be a safe and beneficial option [3,9]. A limitation of our study was a follow-up of only 12 months. Additionally, rhythm assessment was performed using a 12-lead ECG combined with a history of symptoms, but not Holter monitoring. Furthermore, our patients received only standard-of-care examinations postoperatively, and inclusion in this study did not lead to coronary angiography, electrophysiological mapping, or a CT scan to further evaluate surgical success. Finally, sampling bias cannot be excluded, and operation times were quite long in this novel approach, but are expected to be reduced with more experience from the surgical team. Combining minimally invasive approaches that allow for preservation of the sternal bone and thereby reducing patients' discomfort, infection risk, and recovery time is crucial in modern cardiac surgery. Our report shows that the combination of the three procedures can be done feasibly and safely, enabling for future-efficient, cost-effective, and minimally invasive approaches to complex cases. Combining minimally invasive procedures has been performed previously in selected cases, and given that our findings are based on only three cases, the results should be treated with caution.

## Conclusions

Concomitant convergent ablation, thoracoscopic LAA closure, and MIDCAB procedures during a single operation were feasible in the first three cases reported. Furthermore, the combined procedure was safe, with no major postoperative complications and no serious adverse events with 12 months of follow-up. The concomitant procedures may be suitable for selected patients, though the results should be treated with caution as they only present 12 months of follow-up in three cases. Combining minimally invasive procedures can create new approaches for complex patients while still limiting the surgical trauma.
